# Association of epidermal growth factor receptor (EGFR) gene polymorphisms with endometriosis

**DOI:** 10.1097/MD.0000000000015137

**Published:** 2019-04-26

**Authors:** Yu-Mei Wang, Meng-Jun Wu, Yong-Hong Lin, Jie Chen

**Affiliations:** a Chengdu Women's and Children's Central Hospital; b West China Second Hospital of Sichuan University, Chengdu Sichuan, China.

**Keywords:** EGFR, endometriosis, polymorphism, susceptibility

## Abstract

**Aims:**

To investigate the association between polymorphism in the gene encoding the epidermal growth factor receptor (EGFR) and susceptibility to endometriosis among women in southwest China.

**Methods:**

A case-control study involving 201 endometriosis patients and 237 control women without endometriosis was carried out at West China Second Hospital of Sichuan University from June 2016 to December 2017. Two tag single-nucleotide polymorphisms (SNPs) of EGFR gene, rs11977660 and rs2072454 were selected, and the distribution of genotypes and alleles was compared between the 2 groups using the chi-squared test with 2-sided contingency tables.

**Results:**

Genotype at rs11977660 was significantly associated with endometriosis (*P* < .05 for genotype and allele). T/T+C/T genotypes were associated with significantly higher risk of developing endometriosis than the C/C genotype (OR 2.129, 95%CI 1.411–3.212). No significant association was found between genotype at rs2072454 and endometriosis.

**Conclusion:**

Genotypes with a T nucleotide at rs11977660 may significantly increase risk of endometriosis in Chinese.

## 1 Introduction

Endometriosis, a complex disease that is benign but invasive, refers to the ectopic location of endometrium-like glandular epithelium and stroma outside the uterine cavity, including the ovaries, uterosacral ligaments, and pouch of Douglas. Endometriosis usually causes chronic pelvic pain (CPP), dysmenorrhea, pain during or after intercourse, subfertility, and pelvic mass.^[1]^ The etiology and pathogenesis of endometriosis are unclear but appear to involve genetic, endocrine, immune, and environmental factors. Several studies have identified genetic factors associated with risk of endometriosis, which is consistent with the fact that the condition shows familial inheritance patterns and high consistency between monozygotic twins.^[2,3]^

Growth of ectopic endometrial lesions in endometriosis requires new blood vessels, so angiogenesis may be critical in pathogenesis of the disease.^[6]^ Laparoscopy shows that ectopic lesions are surrounded by proliferative vessels, and intra-abdominal angiogenesis appears to be associated with the disease. Indeed, the mediator of vascular angiogenesis, vascular endothelial growth factor (VEGF), has been associated with endometriosis.^[4,5,7]^

Another molecule involved in angiogenesis is epidermal growth factor (EGF) and its cognate epidermal growth factor receptor (EGFR), a tyrosine kinase receptor. EGF stimulates growth of vascular endothelial cells and formation of blood vessels, and its activation affects numerous processes important to cancer development and progression, including cell proliferation, apoptosis, angiogenesis, and metastasis.^[8,9,10]^ Binding of EGF or transforming growth factor-α to EGFR triggers, cell differentiation, and proliferation. EGFR activation stimulates mitotis in the endometrium, which is composed of epithelial, stromal, inflammatory, perivascular, and blood vessel cells, so it plays a central role in endometriosis.

Studies suggest that polymorphism in the EGFR gene, located in chromosomal region 7p12, may affect susceptibility to endometriosis, but results have been inconsistent. A study in South Korea failed to find associations of EGFR single-nucleotide polymorphisms (SNPs) 151904 A>T, 162093 G>A, or 181946 C>T with advanced endometriosis.^[11]^ A similar lack of association was reported for the polymorphisms Egfr+2073 A/T and Egf+61 G/A and risk of endometriosis in a Japanese population.^[12]^ However, a study found evidence of an association of T-containing genotypes and alleles at the EGFR single-nucleotide polymorphism (SNP) 2073∗ with greater risk of endometriosis and leiomyoma.^[13]^

Here, we chose rs11977660 and rs2072454, which are the 2 known common tag SNPs in the EGFR gene, and examined whether 2 SNPs in the EGFR gene may be associated with risk of endometriosis in a Chinese population. We compared genotype and allele distributions between women with or without endometriosis.

## 2 Subjects and methods

This case-control study was carried out in West China Second Hospital of Sichuan University from June 2016 to December 2017. It involved 201 endometriosis patients and 237 healthy women; across both groups, age ranged from 20 to 45 years. Disease was diagnosed based on clinical laboratory results, endoscopy, laparotomy, and pathology. Patients were assigned to stage III or IV according to the revised American Fertility Society (1985) classification. None of the patients had received hormone therapy. The control group included women who underwent surgery for ectopic pregnancy or cesarean section. All subjects in the study were Han Chinese from the southwest part of the country. The study protocol was approved by the Ethics Committee of West China Second Hospital of Sichuan University, and informed consent was obtained from all subjects.

Samples of peripheral venous blood (3 ml) were collected from each subject and stored at 4°C. Within 1 week, genomic DNA was extracted using the Centrifugal Columnar Whole Blood Genomic DNA Extraction Kit (Bioteke Corporation, Beijing, China).

After screening SNPs in the EGFR gene in the database at the US National Center for Biotechnology Information (www.ncbi.nlm.nih.gov/SNP) and in Han Chinese haplotype blocks in the International Hap Map project (www.hapmap.org), we selected the SNPs rs11977660 and rs2072454 for genotyping. To be valid, we stipulated that the pairwise correlation coefficient be >0.8 and the minor allele frequency >20%.

PCR primers for these 2 SNPs were designed based on the GenBank reference sequence (accession no. NT_033968) and synthesized commercially (Invitrogen). The PCR primers for rs11977660 were 5’-ACAAGGCAGTCCAGCAACTT-3’ (forward) and 5’-TCTCTCAAAGTGGATCTGTGATG-3’ (reverse). The primers for rs2072454 were 5’-AAAGAGTGCTCACCGCAGTT-3’ (forward) and 5’-TGCTGAGAAAGTCACTGCTGA-3’ (reverse). Both primers amplified a region of 100 bp.

PCR reactions were performed in a 10-μl reaction volume containing 100 ng genomic DNA, 5 pM of each primer, nuclease-free water, and *Sso*Fast EvaGreen Supermix (Biotium, CA). Reactions were carried out in 96-well unskirted PCR plates with white wells (Bio-Rad Laboratories, CA). The polymerase was activated at 98°C for 3 minutes, then reactions were subjected to 45 cycles of denaturation for 2 seconds at 98°C, followed by annealing for 5 seconds at 60°C. PCR products were heated incrementally in 0.2°C steps from 70°C to 95°C, with a hold time of at least 10 seconds in the melt curve. Data were acquired and analyzed using Precision Melt Analysis Software (Bio-Rad Laboratories, CA). Melting curves were compared between patients and controls in the region from 83.0°C to 88.6°C for rs2072454 and from 75.4°C to 81.3°C for rs11977660. Before comparison, melting curves were subjected to normalization, temperature adjustment and difference steps.

**Figure 1:**
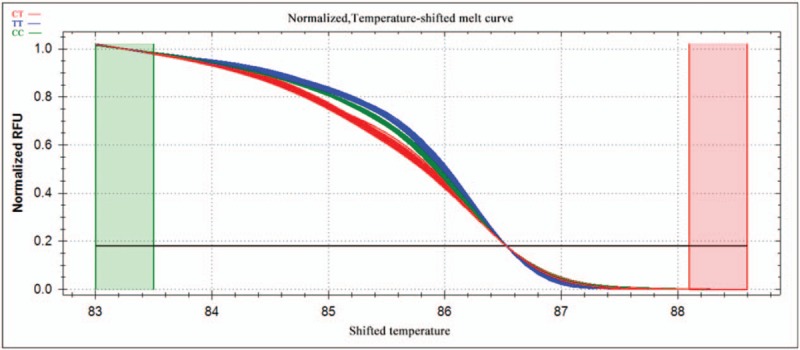
Normalized melting curves for the SNP rs2072454 after temperature shifting. The different colors correspond to the different genotypes as shown at the upper left of the plot. SNP = single-nucleotide polymorphism.

**Figure 2:**
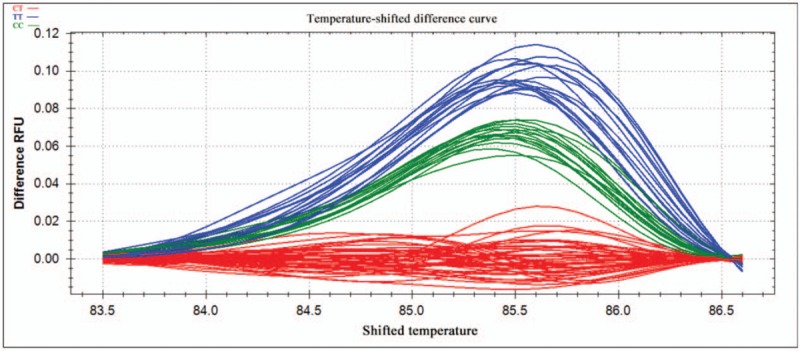
Fluorescence difference curves for the SNP rs2072454 after temperature shifting. The different colors correspond to the different genotypes as shown at the upper left of the plot. The reference curve, CT, was derived from the average fluorescence of all the curves within a selected reference cluster. SNP = single-nucleotide polymorphism.

**Figure 3:**
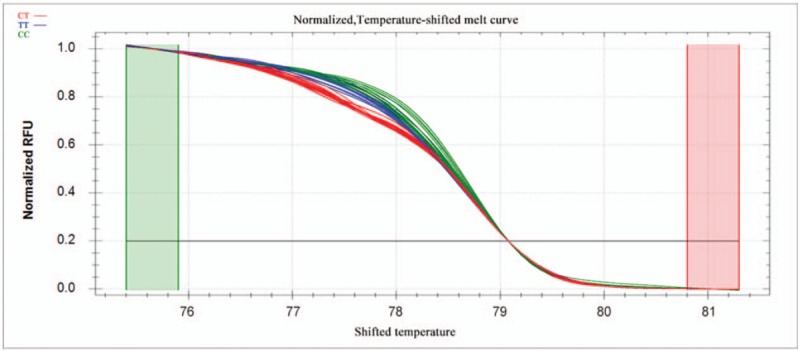
Normalized melting curves for the SNP rs11977660 after temperature shifting. The different colors correspond to the different genotypes as shown at the upper left of the plot. SNP = single-nucleotide polymorphism.

**Figure 4:**
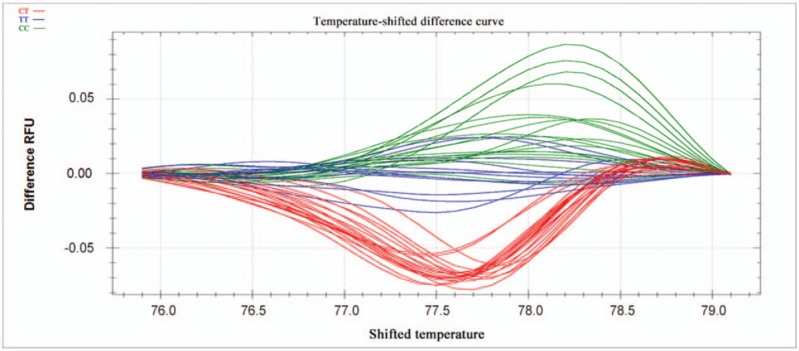
Fluorescence difference curves for the SNP rs11977660 after temperature shifting. The different colors correspond to the different genotypes as shown at the upper left of the plot. The reference curve, TT, was derived from the average fluorescence of all the curves within a selected reference cluster. SNP = single-nucleotide polymorphism.

Normalized curves after temperature shifting are shown in Figures 1 and 3, while the fluorescence differences after subtracting each curve from the reference curve are shown in Figures 2 and 4. Individuals with different homozygous genotypes showed similar melting curves but different melting temperatures. Homozygous individuals were easy to distinguish from one another because one contained an A::T pair and the other, a G::C pair.

Melting curves of heterozygotes differed from those of homozygotes in the low melting region of the transition.

Statistical analysis was carried out using 2-sided probabilities in SPSS 13.0 (IBM, Chicago, IL). The distribution of the EGFR genotypes and alleles was compared between patients and controls using the chi-squared test with 2-sided contingency tables. Unconditional logistic regression was used to assess associations between genotype frequencies and risk of endometriosis in terms of odds ratios (ORs) and 95% confidence intervals (CIs). Differences were considered significant if *P* < .05.

## 3 Results

Patients and controls did not differ significantly in age, weight, height, or history of pregnancy. There was also no significant difference in the distribution of C/T genotypes and allelotypes at rs2072454 (Table 1, *P* > .05). The T/T genotype was not associated with significantly different risk of endometriosis than the C allele (OR 1.442, 95%CI 0.950–2.188; Table 2).

**Table 1:**
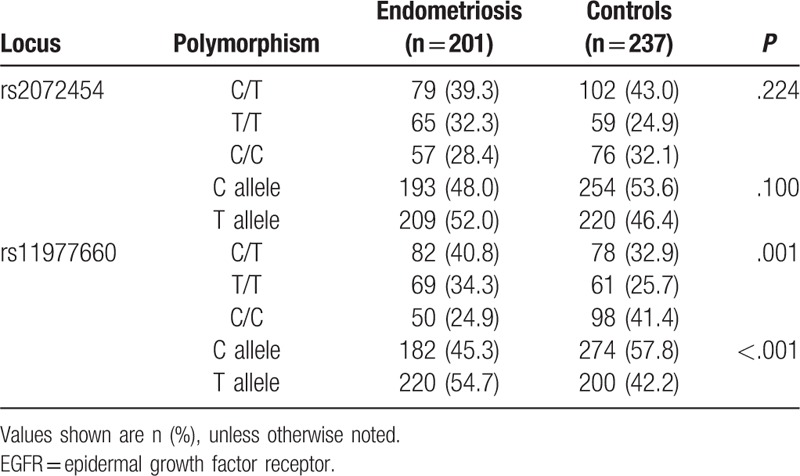
Genotyping of 2 single-nucleotide polymorphisms in the EGFR gene in women from southwest China.

**Table 2:**
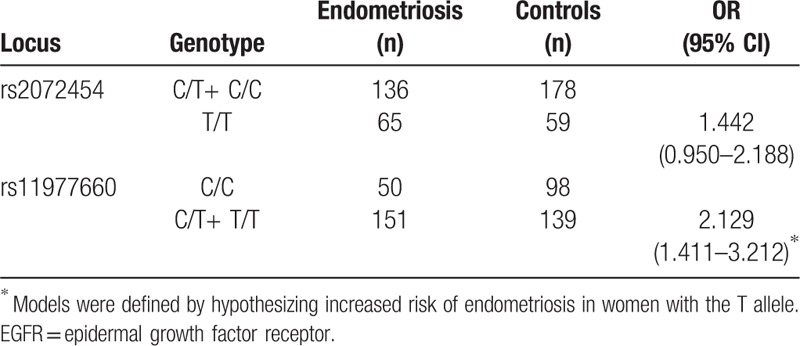
Association of 2 single-nucleotide polymorphisms in the EGFR gene with risk of endometriosis in women from southwest China.

In contrast, frequencies of the 3 C/T genotypes (*P* = .001) and 2 allelotypes (*P* < .001) at rs11977660 were significantly different between patients and controls (Table 1). T/T+C/T genotypes were associated with significantly greater risk of endometriosis than the C/C genotype (OR 2.129, 95%CI 1.411–3.212; Table 2).

## 4 Discussion

Our results suggest that the SNP rs11977660, located in intron 1 of the EGFR gene, is significantly associated with endometriosis in women from southwest China. Endometriosis, although benign, has invasive and metastatic tendencies. It is associated with peritoneal inflammation, fibrosis, formation of adhesions and ovarian cysts. Here we provide evidence of an SNP in the EGFR gene, rs11977660 C/T, that is associated with risk of endometriosis in women in southwest China. Identifying genetic risk factors for the disease may help guide efforts to clarify its pathogenesis, find effective treatments, reduce recurrence and mitigate its damage.

Our results, together with those of another study^[13]^ linking the EGFR 2073∗ polymorphism to risk of endometriosis, may begin to clarify genetic risk factors behind this disease. Our results are consistent with the idea that EGFR expression or activity is associated with occurrence and progression of endometriosis. For example, 1 study^[14]^ showed that 50% of ectopic endometrial epithelium expresses EGFR. Polymorphism in the EGFR gene such as rs11977660 C/T may alter the expression or activity of the EGFR, which should be examined in future work. Differences in EGFR levels or levels may help explain individual differences in endometriosis susceptibility.

We performed high-resolution melting analysis of 100-bp amplicons to genotype SNPs in our population. This amplicon length was long enough for robust hybridization but sufficiently short to reliably measure differences in melting temperature among the different genotypes. An advantage of high-resolution melting is that multiple SNPs can be screened to define haplotype structures associated with the disease. Future work should examine integrating several SNPs into a combined model, which may be more effective than the single-SNP approach in the present study. In such work, the HapMap project (http://www.hapmap.org) may prove powerful for illuminating the range of genetic polymorphisms linked to pathogenesis of endometriosis.

Our results suggest that, at least in Chinese women, the EGFR SNP rs11977660 may be a useful marker for determining genetic susceptibility to endometriosis. Angiogenesis may be a critical process in the pathogenesis of developing endometriosis, so large studies are needed to investigate the efficacy of anti-angiogenic therapy.

## Author contributions

**Conceptualization:** Lin Yonghong, Chen Jie.

**Data curation:** Wu Mengjun.

**Resources:** Wang Yumei.

**Writing – review & editing:** Chen Jie.

## References

[1] Nnoaham KE Hummelshoj L Webster P Impact of endometriosis on quality of life and work productivity: a multicenter study across ten countries. Fertil Steril 2011;96:366–73. 21718982 10.1016/j.fertnstert.2011.05.090 PMC3679489

[2] Simpson JL Bischoff FZ Kamat A Genetics of endometriosis. Obstet Gynecol Clin North Am 2003;30:21–40. 12699256 10.1016/s0889-8545(02)00051-7

[3] Barlow DH Kennedy S Endometriosis: new genetic approaches and therapy. Annu Rev Med 2005;56:345–56. 15660516 10.1146/annurev.med.55.091902.103805

[4] Szczepańska M Mostowska A Wirstlein P Involvement of vascular endothelial growth factor -460 C/T, +405 G/C and +936 C/T polymorphisms in the development of endometriosis. Biomed Rep 2015;3:220–4. 26075076 10.3892/br.2014.409 PMC4448009

[5] Cardoso JV Abrão MS Vianna-Jorge R Combined effect of vascular endothelial growth factor and its receptor polymorphisms in endometriosis: a case-control study. Eur J Obstet Gynecol Reprod Biol 2017;209:25–33. 27836223 10.1016/j.ejogrb.2016.10.046

[6] Deguchi M Ishiko O Sumi T Expression of angiogenic factors in extrapelvic endometriosis. Oncol Rep 2001;8:1317–9. 11605057 10.3892/or.8.6.1317

[7] Kim SH Choi YM Choung SH Vascular endothelial growth factor gene +405 C/G polymorphism is associated with susceptibility to advanced stage endometriosis. Hum Reprod 2005;20:2904–8. 15979997 10.1093/humrep/dei146

[8] Yarden Y Sliwkowski MX Untangling the ErbB signaling network. Nat Rev Mol Cell Biol 2001;2:127–37. 11252954 10.1038/35052073

[9] Arteaga CL Overview of epidermal growth factor receptor biology and its role as therapeutic target in human neoplasia. Semin Oncol 2002;29:3–9. 10.1053/sonc.2002.35642 12422308

[10] Jorissen RN Walker F Pouliot N Epidermal growth factor receptor: mechanisms of activation and signaling. Exp Cell Res 2003;284:31–53. 12648464 10.1016/s0014-4827(02)00098-8

[11] Lee GH Choi YM Kim JM Association of epidermal growth factor receptor gene polymorphisms with advanced endometriosis in a Korean population. Eur J Obstet Gynecol Reprod Biol 2012;164:196–9. 22770632 10.1016/j.ejogrb.2012.06.004

[12] Inagaki M Yoshida S Kennedy S Association study between epidermal growth factor receptor and epidermal growth factor polymorphisms and endometriosis in a Japanese population. Gynecol Endocrinol 2007;23:474–8. 17852426 10.1080/09513590701521057

[13] Hsieh YY Chang CC Tsai FJ T homozygote and allele of epidermal growth factor receptor 2073 gene polymorphism are associated with higher susceptibility to endometriosis and leiomyomas. Fertil Steril 2005;83:796–9. 15749523 10.1016/j.fertnstert.2004.08.032

[14] Prentice A Epidermal growth factor receptor expression in normal endometrium and endometriosis: an immunohistochemical study. Br J Obster Gynecol 1992;99:395. 10.1111/j.1471-0528.1992.tb13756.x 1622912

